# PEDOT: PSS promotes neurogenic commitment of neural crest-derived stem cells

**DOI:** 10.3389/fphys.2022.930804

**Published:** 2022-08-17

**Authors:** Alessandra Pisciotta, Alice Lunghi, Giulia Bertani, Rosanna Di Tinco, Laura Bertoni, Giulia Orlandi, Fabio Biscarini, Michele Bianchi, Gianluca Carnevale

**Affiliations:** ^1^ Department of Surgery, Medicine, Dentistry and Morphological Sciences with Interest in Transplant, Oncology and Regenerative Medicine, University of Modena and Reggio Emilia, Modena, Italy; ^2^ Center for Translational Neurophysiology of Speech and Communication, Fondazione Istituto Italiano di Tecnologia, Ferrara, Italy; ^3^ Sezione di Fisiologia, Università di Ferrara, Ferrara, Italy; ^4^ Department of Life Sciences, Università di Modena e Reggio Emilia, Modena, Italy

**Keywords:** conductive polymers, nanostructured thin films, dental pulp stem cells, cell differentiation, stemness, immunomodulatory properties

## Abstract

Poly (3,4-ethylendioxythiophene) polystyrene sulphonate (PEDOT:PSS) is the workhorse of organic bioelectronics and is steadily gaining interest also in tissue engineering due to the opportunity to endow traditional biomaterials for scaffolds with conductive properties. Biomaterials capable of promoting neural stem cell differentiation by application of suitable electrical stimulation protocols are highly desirable in neural tissue engineering. In this study, we evaluated the adhesion, proliferation, maintenance of neural crest stemness markers and neurogenic commitment of neural crest-derived human dental pulp stem cells (hDPSCs) cultured on PEDOT:PSS nanostructured thin films deposited either by spin coating (SC-PEDOT) or by electropolymerization (ED-PEDOT). In addition, we evaluated the immunomodulatory properties of hDPSCs on PEDOT:PSS by investigating the expression and maintenance of the Fas ligand (FasL). We found that both SC-PEDOT and ED-PEDOT thin films supported hDPSCs adhesion and proliferation; however, the number of cells on the ED-PEDOT after 1 week of culture was significantly higher than that on SC-PEDOT. To be noted, both PEDOT:PSS films did not affect the stemness phenotype of hDPSCs, as indicated by the maintenance of the neural crest markers Nestin and SOX10. Interestingly, neurogenic induction was clearly promoted on ED-PEDOT, as indicated by the strong expression of MAP-2 and β —Tubulin-III as well as evident cytoskeletal reorganisation and appreciable morphology shift towards a neuronal-like shape. In addition, strong FasL expression was detected on both undifferentiated or undergoing neurogenic commitment hDPSCs, suggesting that ED-PEDOT supports the expression and maintenance of FasL under both expansion and differentiation conditions.

## Introduction

Conductive polymers are steadily gaining interest in bioengineering and regenerative medicine due to their unique physico-chemical properties, easy processability and the intriguing possibility to endow traditional biomaterials with conductive properties. The latter can be exploited for instance to record electrophysiological signals, locally apply current stimuli or deliver drugs, ultimately increasing the functional and biological performance of pristine materials (Simon et al., 2016; Guex et al., 2017; Arbring Sjöström et al., 2018). Among these, poly (3,4ethylendioxythiopene) polystyrene sulfonate (PEDOT:PSS) carved out a predominant role as an active material in a number of bio-applications, including wearable and stretchable biosensors, neural arrays for *in vitro* and *in vivo* applications up to electroactive implantable scaffolds (Balint et al., 2014; Guex et al., 2017; Fan et al., 2019; Bianchi et al., 2022). The success of PEDOT:PSS can be attributed to its excellent electrical properties, i.e., high conductivity (especially upon secondary doping) (Takano et al., 2012), high charge storage capacitance (Bianchi et al., 2020) and mixed ionic/electronic conductance (Rivnay et al., 2016). In addition, PEDOT:PSS can be easily incorporated into biocompatible hydrogels, yielding ultra-soft conductive electrodes and substrates for highly stable and better integrated biointerfaces, characterised by improved cell attachment and viability and decreased foreign body reaction (Ferlauto et al., 2018; Liu et al., 2018; Lu et al., 2019).

Overall, PEDOT:PSS is a commercially available commodity. Commercial aqueous dispersions with different degrees of conductivity and biocompatibility enable the manufacturing of a wide range of organic electronic devices and 3D constructs by low cost, reproducible wet processes, including 3D printing, inkjet printing, screen printing and roll-to-roll printing (Fan et al., 2014; Liu et al., 2015; Zielke et al., 2018; Di Lauro et al., 2020; Calandra; Yuk et al., 2020; Sebastianella et al., 2021). Besides, PEDOT:PSS coatings and thin films can be deposited by electrochemical polymerisation on conductive substrates, even micrometer-sized ones, such as on microelectrodes used for neural recording (Asplund et al., 2009; Bodart et al., 2019; Boehler et al., 2019; Carli et al., 2019; Lunghi et al., 2022). A clear advantage of electrodeposited PEDOT:PSS over aqueous formulations is that the biocompatibility and functional properties of the polymer can be easily customised by replacing the PSS^−^ counterion with alternative dopants, including carbon nanotubes, biomolecules (e.g., DNA) and drugs (e.g., anti-inflammatory) (Kim et al., 2002; Kozai et al., 2016; Wang et al., 2017; Tekoglu et al., 2020; Fenoy et al., 2021; Guzzo et al., 2021).

Interestingly, PEDOT-based electroactive materials have already been shown to stimulate cell adhesion, proliferation and differentiation, finding application mainly in bone and neural tissue engineering (Guex et al., 2017; Neo et al., 2017; Jayaram et al., 2019; Iandolo et al., 2020; Magaz et al., 2020). A particularly intriguing aspect is the possibility of promoting stemness properties including neural stem cells differentiation, by applying appropriate low-voltage stimulation trains (Neo et al., 2017; Sordini et al., 2021; Pires et al., 2015; Šafaříková et al., 2018). However, our current understanding of the biological response of neural stem cells to PEDOT-based electroactive materials is still in its infancy. It is worth mentioning, available *in vitro* studies investigating the effects of PEDOT: PSS-based materials on neural differentiation were conducted using different cell sources, including transformed neuronal cell lines or mesenchymal stem cells, which embryologically might not fully encompass the properties of neural tissues (Neo et al., 2017; Gordon et al., 2013).

Based on these premises, neural crest derived stem cells represent a promising cell source to investigate the interactions between PEDOT: PSS and stem cells. As well established, the neural crest can be considered as the fourth germ layer, which originates during the third week of embryonic development in parallel with neural tube formation. Neural crest migrating cells then give rise to most of the craniofacial tissues, including dental pulp, neural, connective tissues and Schwann cells (Chai et al., 2000; Janebodin et al., 2011; Shyamala et al., 2015). Among human dental pulp cells, a purer stem cell population was identified and extensively characterised for its peculiar features related to neural crest, i.e., regenerative potential with functional recovery associated with a reduction of fibrosis, and immunomodulatory properties *in vitro* and *in vivo* (Bianchi et al., 2017; Pisciotta et al., 2018; Zordani et al., 2019; Pisciotta et al., 2020; Di Tinco et al., 2021). In particular, due to their embryonic neural crest origin, human dental pulp stem cells (hDPSCs) have emerged as ideal cell sources for neuroscience studies (Granz and Gorji, 2020; Heng et al., 2022). It has been demonstrated that hDPSCs are able to secrete neuroprotective growth factors, such as nerve growth factor (NGF), brain-derived neurotrophic factor (BDNF) and neurotrophin 3 (NT-3) (Kolar et al., 2017; Carnevale et al., 2018). *In vitro* and *in vivo* studies proved that hDPSCs exert such neuroprotective effects allowing to promote axon regeneration and neurite outgrowth, to reduce the neurodegeneration during the early phases of neural apoptosis and support sensory neuron survival (Nosrat et al., 2001; Nicola et al., 2017; Sultan et al., 2020).

Previous studies have also shed light on the need to combine biomaterials with dental pulp stem cells in order to improve cell engraftment and favour a functional integration into the recipient neural tissue (Luzuriaga et al., 2021). To this purpose, it is well established that the ideal biomaterial for multiple regenerative purposes is expected to maintain the stem properties of the cells involved in the regenerating processes, which indeed includes self-renewal and differentiation capabilities and, likewise importantly, the control of host immune response towards biomaterials.

Therefore, given the strong interest into deepening our knowledge about stem properties of dental pulp stem cells when growing and differentiating on a conductive polymeric substrate, we explored in this study for the first time the behaviour of neural crest-derived hDPSCs cultured on PEDOT: PSS substrates. In particular, we investigated the cellular response of hDPSCs cultured onto two main types of PEDOT: PSS thin films, obtained either by spin coating from aqueous dispersion or by electrochemical polymerisation, as the fabrication approach may significantly affect the cellular response (Bystrenova et al., 2008; Dionigi et al., 2010; Tonazzini et al., 2010; Valle et al., 2010). We specifically focused on the ability of the PEDOT:PSS thin films to support the proliferation and commitment of hDPSCs to the neurogenic lineage and to maintain the immunomodulatory properties of these neural crest derived stem cells, which further widen their stemness phenotype.

## Materials and methods

### Materials

PEDOT: PSS dispersion was purchased from Sigma-Aldrich (Product number: 483095, CAS number 155090-83-8, Sigma-Aldrich, MO, United States). The nominal solid content and the PEDOT to PSS ratio of the PEDOT: PSS dispersion were 1.3% and 1:1.6 by weight, respectively. (3-Glycidyloxypropyl) trimethoxysilane (GOPS, Sigma-Aldrich, MO, United States) was used as received without further purification. 3,4-Ethylenedioxythiophene (EDOT) and polysulfate sodium (NaPSS) were obtained from Sigma Aldrich (MO, United States) and used to obtain the solution (EDOT 0.01 M; NaPSS 0.8% w/w) for the electrochemical deposition of PEDOT: PSS. Borosilicate glass slides (thickness ∼1 mm, Thermo Scientific, MA, United States) were used as substrate for the deposition of the PEDOT: PSS films by spin coating after ultrasonic cleaning in a mixed solution of pure ethanol, isopropanol and milliQ water (1:1:1 by volume) and drying under sustained flux of pure nitrogen. Fluorine Tin Oxide (FTO) slabs (1.5 × 1 cm^2^, thickness ∼1 mm) were used as conductive substrate for the electrochemical polymerisation of PEDOT:PSS films. FTO slabs were used after ultrasonic cleaning in a solution of pure ethanol and milliQ water (1:1), drying under sustained flux of pure nitrogen and a heating step at 450°C for 30 min in order to clean the surface from organic residues and after cooling overnight in the muffle.

### Preparation of PEDOT: PSS films

Spin-coated PEDOT:PSS thin films (from here onwards SC-PEDOT) were obtained on borosilicate glass slides by spinning a 30 μl of a mixture of PEDOT:PSS and GOPS (0.2% v/v) according to the following protocol: 10 s at 300 rpm, 20 s at 600 rpm and 20 s at 2000 rpm (Bianchi et al., 2020). Then, films were baked at 120°C for 45 min to allow water complete evaporation and film consolidation. For electrical measurements, a thin Au layer (20 nm) was preliminary sputtered on the glass substrate. Electrodeposited PEDOT:PSS thin films (from here onwards ED-PEDOT) were obtained on FTO substrates (the working electrode) by using a large area platinum mesh (30 × 15 mm^2^) as counter electrode and a standard Ag|AgCl electrode (3 M KCl) as reference. A solution of EDOT, 0.01 M in aqueous NaPSS (0.7% w/w) was used for the electrochemical polymerisation obtained by sweeping the potential between 0 and +1 V for 10 cycles.

### Surface characterization of PEDOT: PSS films

Atomic Force Microscopy (AFM) was used to analyse surface topography of the spun coated and electrodeposited PEDOT:PSS thin films. Images were acquired in air at room temperature using a Park XE7 AFM System (Park System, Suwon, Korea) operating in non-contact mode. Pre-mounted silicon cantilever with Al backside reflecting coating, typical tip curvature radius ∼7 nm, k ∼ 26 N m^−1^ and resonance frequency ∼300 kHz were used (OMCL-AC160TS, Olympus Micro Cantilevers, Tokyo, Japan). The RMS of the films was extracted from several topography images acquired at different scan sizes (from 500 nm to 5 μm) using the Park Systems XEI Software (Park Systems, Suwon, Korea). Contact angle measurements were carried out to evaluate the wettability of the PEDOT:PSS thin films using a home-built contact angle measurement unit. The value of the water contact angle was obtained by averaging several measurements of the left and right contact angle of a milliQ water drop deposited on at least three different areas of the sample surface. Images were analysed with the ImageJ free software (https://imagej.nih.gov/).

### Electrical and electrochemical characterization of PEDOT: PSS films

Sheet resistance of SC-PEDOT and ED-PEDOT was obtained by means of four-point probe measurements [ref]. Briefly, a 1 × 1 cm^2^ area was delimited on the samples, then four blunt stainless steel probes were placed in contact with the sample surface at the vertices of the square. The current was streamed along the sample by a two channel Keysight B2912A source-measure unit (Keysight, CA, United States) so that a constant value of current was streamed for 10 s before it was raised to the desired value (from 0.1 to 0.5 mA).

Sheet resistance (*Rs*) was calculated with the following equation:

$$Rs=\frac{\left(R*\pi \right)}{ln2}$$

where R is the resistance extracted from the first Ohm law. For measurements in hydrated conditions, samples were soaked for 1 h in milliQ water before electrical measurements.

Electrochemical Impedance Spectroscopy (EIS) was used to preliminary investigate the electrochemical properties of SC-PEDOT and ED-PEDOT. EIS measurements were carried out in a three-electrodes cell using a standard Ag|AgCl (3 M KCl) electrode as reference electrode and a large area platinum mesh (30 × 15 mm^2^) as counter electrode. SC-PEDOT and ED-PEDOT substrates were used as working electrodes. All measurements were performed in saline solution (NaCl 0.15 M) over the following frequency range: 0.1–10^5^ Hz.

### Human dental pulp stem cells: cell isolation and immune selection

The study was carried out in compliance with the recommendations of Comitato Etico Provinciale-Azienda Ospedaliero-Universitaria di Modena (Modena, Italy), which provided the approval of the protocol (ref. number 3299/CE; 5 September 2017). Human dental pulp samples were harvested from third molars of adult subjects (*n* = 3; 18–25 years), undergoing routine dental extraction, who gave their written informed consent, in accordance with the Declaration of Helsinki. Cells were isolated from human dental pulp as formerly described (Pisciotta et al., 2020). Briefly, dental pulp was collected from the teeth and underwent enzymatic digestion through incubation in a digestive solution, i.e., α-MEM containing 3 mg/ml type I collagenase plus 4 mg/ml dispase, for 1 h at 37°C and 5% CO_2_. Digested pulp was then filtered onto 100 µm Falcon Cell Strainers in order to obtain a cell suspension, which was then plated in 25 cm^2^ culture flasks and expanded in standard culture medium (α-MEM supplemented with 10% heat inactivated foetal bovine serum (FBS), 2 mM L-glutamine, 100 U/ml penicillin, 100 μg/ml streptomycin; all from Sigma Aldrich, St. Louis, MO, United States) at 37°C and 5% CO_2_. Following cell expansion, dental pulp cells were immune-selected by using MACS® separation kit, according to manufacturer’ instructions. In order to sort a purer dental pulp stem cells (hDPSCs) population two sequential immune selections were performed by using mouse IgM anti-STRO-1 and IgG rabbit anti-c-Kit primary antibodies (Santa Cruz Biotechnology, Dallas, TX, United States) (Di Tinco et al., 2021a). The following magnetically labelled secondary antibodies were used: anti-mouse IgM and anti-rabbit IgG (Miltenyi Biotec, Bergisch Gladbach, Germany). Human DPSCs expressing STRO-1 and c-Kit were expanded in standard culture medium and passaged when reaching 70% confluency, cells at passage three were used for each experimental evaluation.

### Immunophenotypical characterization and *in vitro* multi-lineage differentiation of STRO-1^+^/c-Kit^+^ hDPSCs

In order to confirm the phenotype of the immune-selected hDPSCs the expression of the stemness markers STRO-1 and c-Kit, the neural crest-related antigens nestin and SOX10, and the immunomodulatory molecule FasL was investigated. Subsequently, the multipotency of the STRO-1^+^/c-Kit^+^ hDPSCs population was demonstrated by culturing cells in appropriate differentiation media to reach osteogenic, myogenic and glial differentiation, respectively, as formerly described (Di Tinco et al., 2021a; Zordani et al., 2019; Carnevale et al., 2018). At the end of each differentiation experiment, the commitment was evaluated by assessing the expression of lineage related markers with the use of the following primary antibodies: mouse anti-osteocalcin (OCN), rabbit anti-RUNX2 (Abcam), mouse anti-myogenin, rabbit anti-desmin and rabbit anti-S100b (all from Sigma Aldrich). Confocal immunofluorescence analyses were performed as detailed below.

### Cell adhesion, cell proliferation and morphology

Human STRO-1^+^/c-Kit^+^ DPSCs were seeded (3,000 cells/cm^2^) on SC and ED-PEDOT coated surfaces in 6-multi-well culture plates and kept in standard culture medium for 1 week. After 16, 24, 48 h and 5 days of culture in standard expansion medium, hDPSCs were fixed in ice-cold 4% paraformaldehyde in phosphate buffer saline (PBS) for 20 min at room temperature. Then, after rinsing with PBS cells were permeabilized with 0.1% Triton X-100 in PBS and subsequently labelled with Alexa-546 conjugated Phalloidin (Abcam, Cambridge, United Kingdom) for 1 h at room temperature. Subsequently, nuclei were stained with 1 μg/ml 4′,6-diamidino-2-phenylindole (DAPI) in PBS. PEDOT-hDPSCs samples were mounted with FluoroMount anti-fading medium (Thermo Fisher Scientific, Waltham, MA, United States) on glass cover slides. Cell proliferation was evaluated by counting the DAPI-labelled nuclei on five randomly selected fields measuring 2.5 × 10^5^ μm^2^ on three samples for each experimental group by a blind operator and acquired by using a Nikon A1 confocal fluorescence microscope (Nikon, Tokyo, Japan), as formerly described (Di Tinco et al., 2021b). hDPSCs cultured on plastic culture plates were used as controls.

### Evaluation of stemness and neural crest markers in hDPSCs cultured on PEDOT: PSS thin films

After 2 and 7 days of culture on SC- and ED-PEDOT coated surfaces, the expression of the stemness markers STRO-1 and c-Kit and the neural crest markers Nestin and SOX10 was evaluated in hDPSCs, by confocal immunofluorescence analyses. Briefly, cells were fixed in ice-cold 4% paraformaldehyde in phosphate buffer saline (PBS) for 20 min at room temperature, then rinsed with PBS, permeabilized with 0.1% Triton X-100 in PBS if necessary and, after blocking with 3% bovine serum albumin (BSA) in PBS, were incubated with the following primary antibodies: mouse IgM anti-STRO1, rabbit IgG anti-c-Kit (Santa Cruz, Dallas, TX, United States), mouse anti-nestin (Merck Millipore, Burlington, MA, United States) and rabbit anti-SOX10 (Abcam, Cambridge, United Kingdom), all diluted 1:100 in 1% BSA in PBS, for 1 h at room temperature. After rinsing thrice with 1% BSA in PBS, primary antibodies were revealed by using the following secondary antibodies: goat anti-mouse IgM AlexaFluor 488, goat anti-rabbit AlexaFluor 546 (Thermo Fisher Scientific), all diluted 1:200 in 1% BSA in PBS, for 1 h at room temperature. Finally, nuclei were stained with 1 μg/ml DAPI, then samples were mounted with Fluoromount. The multi-labeling immunofluorescence experiments were carried out avoiding cross-reactions between primary and secondary Abs. Confocal imaging was carried out with a Nikon A1 confocal laser scanning microscope. The confocal serial sections were processed with Fiji ImageJ software (NIH, Bethesda, MD, United States) to obtain 3-dimensional projections and image rendering was performed by Adobe Photoshop Software, as previously described (Di Tinco et al., 2021a).

### Neurogenic induction of hDPSCs on PEDOT: PSS thin films

STRO-1^+^/c-Kit^+^ hDPSCs were seeded at 6,000 cells/cm^2^ on the PEDOT coated surfaces in 6-multiwell plates and kept in standard culture medium upon cell adhesion. Then, medium was replaced with neurogenic medium, as described in a previous study (Pisciotta et al., 2018). Neurogenic conditioning was performed for 1 and 3 weeks and the differentiation medium was changed twice a week. After 7 days of induction, hDPSCs were assayed for the expression of neuronal differentiation markers through confocal immunofluorescence analysis, by using the following primary antibodies: mouse anti-MAP-2 (Sigma-Aldrich, Saint Louis, MO, United States) and rabbit anti-β-Tubulin-III (Cell Signaling Technology, Danvers, MA, United States).

The achievement of neuronal commitment was further investigated after 3 weeks of induction, by means of Real Time PCR analysis of lineage specific markers. Briefly, human DPSCs were homogenised, total RNA was extracted and purified using the PureLink RNA columns (Thermo Fisher Scientific). cDNA synthesis was carried out with Maxima First Strand cDNA Synthesis Kit with DNase I treatment (Thermo Fisher Scientific). Quantitative real-time PCRs were performed using SYBR Green Master mix (Bio-Rad Laboratories) on CFX Connect Real-time PCR instrument (Bio-Rad Laboratories), by using the following oligonucleotides: human RPLP0 (F: TAC ACC TTC CCA CTT GCT GA, R: CCA TAT CCT CGT CCG ACT CC); human β-Tubulin-III (F: TCA​GCG​TCT​ACT​ACA​ACG​AGG​C, R: GCC​TGA​AGA​GAT​GTC​CAA​AGG​C) human MAP-2 (F: AGG​CTG​TAG​CAG​TCC​TGA​AAG​G, R: CTT​CCT​CCA​CTG​TGA​CAG​TCT​G), human RBFOX3 (NeuN) (F: TAC​GCA​GCC​TAC​AGA​TAC​GCT​C, R: TGG​TTC​CAA​TGC​TGT​AGG​TCG​C), human Synaptophysin (hSYP) (F: TCG​GCT​TTG​TGA​AGG​TGC​TGC​A, R: TCA​CTC​TCG​GTC​TTG​TTG​GCA​C).

Relative quantification was calculated from the ratio between the cycle number (Ct) at which the signal crossed a threshold set within the logarithmic phase of the given gene and that of the reference hRPLP0. Mean values of the duplicate results of three independent experiments for each sample were used as individual data for 2^−ΔΔCt^ statistical analysis. hDPSCs differentiated in neurogenic medium on plastic culture plates were used as controls.

### Evaluation of fas ligand expression

The expression of FasL was evaluated in hDPSCs after 7 days of neurogenic induction on both SC- and ED-PEDOT films. Immunofluorescence analysis was carried out by using a primary rabbit anti-FasL antibody (1:100; Santa Cruz Biotechnology) and revealed by a secondary goat anti-rabbit Alexa488 (1:200; Thermo Fisher Scientific), as previously described (Pisciotta et al., 2018). Undifferentiated hDPSCs cultured in standard expansion medium were used as controls. Immunolabeling intensity of FasL expression in all the experimental groups was quantified through pseudocolour analysis: blue to white arrays the colours in a spectrum with blue assigned to a lower value than white (Pisciotta et al., 2020).

### Statistical analysis

All the experiments were performed in triplicate. Data were expressed as mean ± standard deviation (SD). One way ANOVA followed by Newman-Keuls post hoc test was performed to analyse differences among three or more experimental groups. Differences between two groups were analysed by Student t test (GraphPad Prism Software version eight Inc., San Diego, CA, United States). In any case, statistical significance was set for *p* < 0.05.

## Results

### Characterization of the PEDOT: PSS films

**FIGURE 1:**
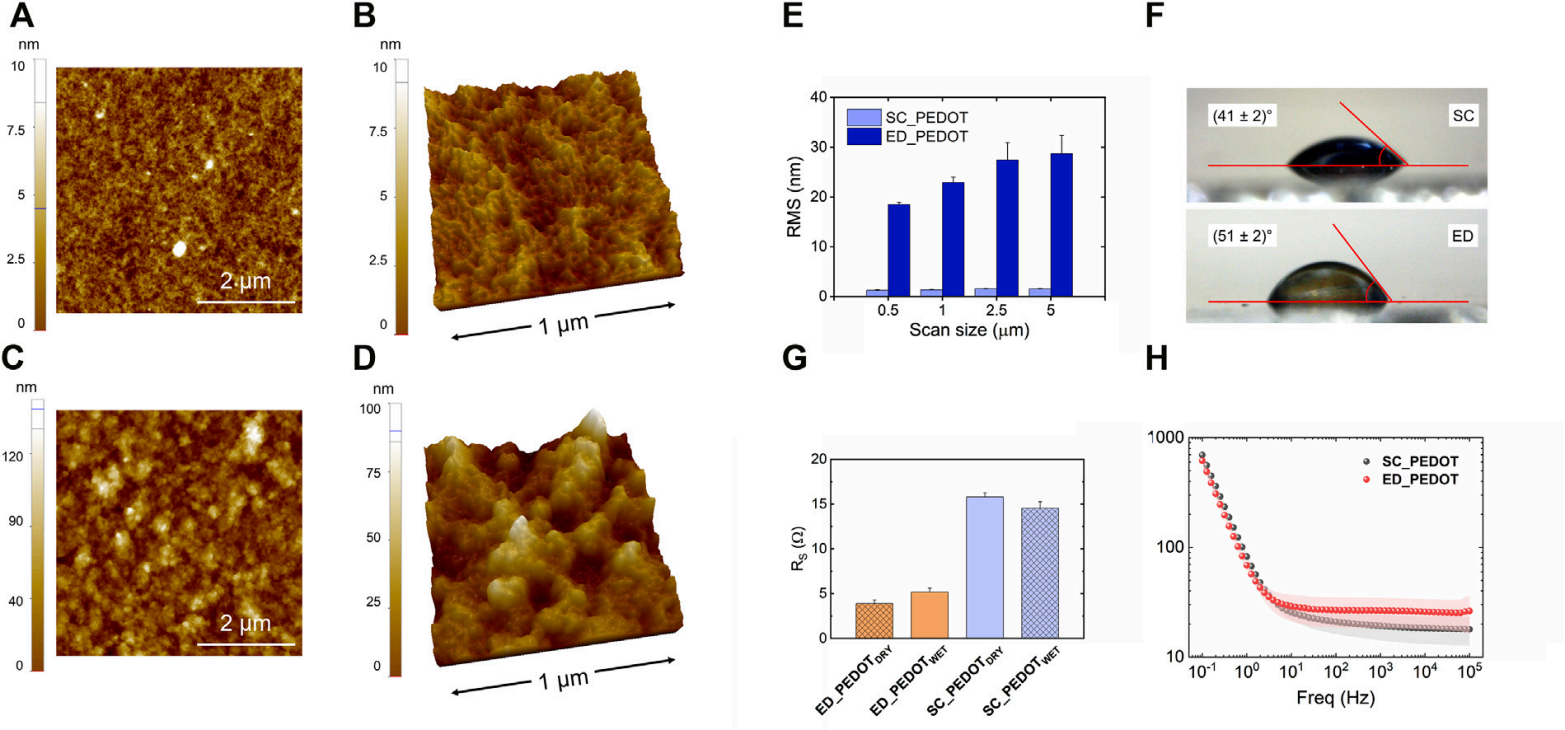
Surface characterization of SC-PEDOT and ED-PEDOT films. 2D **(A,C)** and 3D **(B,D)** AFM topography images of SC-PEDOT **(A,B)** and ED-PEDOT **(C,D)** films underlying the nanostructured granular surface; **(E)** RMS roughness of both the films acquired at different length scales; **(F)** water contact angle values obtained on both the films; **(G)** dry and wet sheet resistance of SC-PEDOT and ED-PEDOT; **(H)** Bode |Z| plot of SC-PEDOT and ED-PEDOT.

The surface topography of spin-coated (SC-PEDOT, thickness: 88 ± 5 nm) and electrodeposited (ED-PEDOT, thickness: 50 ± 8 nm) thin films was analysed by AFM (Figure 1). Spin-coated films showed a much smoother surface than electrodeposited ones (Figures 1A–D), as previously reported (Carli et al., 2019; Bianchi et al., 2020). Accordingly, the RMS roughness of ED-PEDOT was steadily higher than that of SC-PEDOT (from 15 to 20 times in the considered image scan size, Figure 1E). The surface of both PEDOT films was characterised by the presence of nanograins, those of ED-PEDOT being larger (lateral size: 65 ± 4 nm) than those of SC-PEDOT (lateral size: 36 ± 3 nm). This difference can be ascribed to the fact that during electropolymerization, small oligomers of PEDOT:PSS nucleate, coalesce and grow in size on the surface of the conductive substrate, achieving dimensions generally larger than 50 nm (Subramanian & Martin, 2021). On the contrary, the spin coating process casts on the substrate the PEDOT:PSS nanoclusters (average size <50 nm) already present in the starting aqueous dispersion (Posudievsky et al., 2014), sometimes giving rise to nanofibrillar structures under certain conditions or when additives are used (Thomas et al., 2014; Bianchi et al., 2020). The surface wettability of PEDOT:PSS films is shown in Figure 1F. The rougher surface of the electrodeposited films resulted in a marginally more hydrophobic surface compared to the spin-coated ones. To be noted, the water contact angle of both the PEDOT:PSS thin films was in the range 40° ÷ 50°, thus well within the regime generally considered suitable for cell adhesion (Valle et al., 2010; Ventre et al., 2012; Cai et al., 2020). Electrical and electrochemical properties are figures of merit for conductive polymers. Therefore, although here we did not apply any potential differences to our conductive substrates during cellular experiments, we preliminary analysed both the sheet resistance and the electrochemical impedance of the PEDOT:PSS films to provide a broader characterization of the substrates investigated. Figure 1G shows the sheet resistance of PEDOT:PSS films in both dry and hydrated conditions. As can be observed, the sheet resistance did not vary significantly under both dry and hydrated conditions and was only slightly different between SC-PEDOT and ED-PEDOT, being the Rs values in the 5–15 Ω range. The results of the electrochemical impedance spectroscopy analysis are shown in Figure 1H. The Bode plot indicates that the impedance moduli of SC-PEDOT and ED-PEDOT were almost superimposable at all investigated frequencies, both showing a large frequency-independent region at high frequencies for both samples, benchmark characteristic of PEDOT:PSS coatings. Overall, these results indicate that the SC-PEDOT and ED-PEDOT films investigated in this study showed similar electrical and electrochemical properties.

### Immune phenotype and multi-lineage differentiation potential of STRO-1^+^/c-Kit^+^ hDPSCs

**FIGURE 2:**
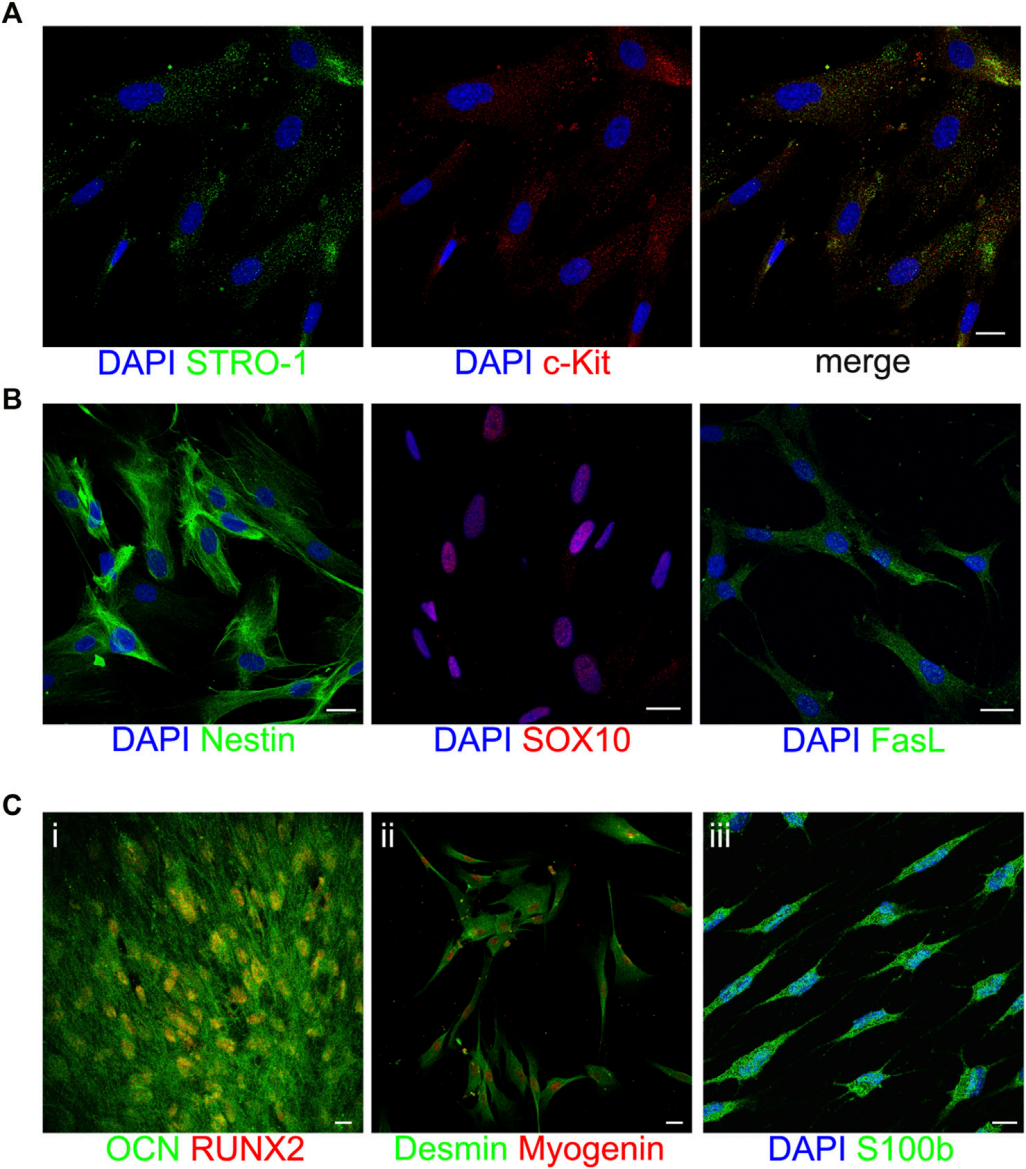
Characterization of STRO-1^+^/c-Kit^+^ hDPSCs. Representative confocal fluorescence images showing the expression of the stemness markers STRO-1 and c-Kit **(A)**, the neural crest markers Nestin and SOX10 and the immunomodulatory molecule FasL **(B)** in immune-selected hDPSCs. Multilineage differentiation potential of STRO-1^+^/c-Kit^+^ hDPSCs is shown by the expression of osteogenic, myogenic and glial specific markers **(C)**. Nuclei were counterstained with DAPI (blue). Scale bar: 10 μm.

The stem phenotype of immune-selected hDPSCs was demonstrated by the expression of the typical stemness markers STRO-1 and c-Kit (Figure 2A), the neural crest related antigens Nestin and SOX10, as well as the immunomodulatory molecule FasL (Figure 2B). As reported in Figure 2C, STRO-1^+^/c-Kit^+^ hDPSCs also proved their capability to commit into osteogenic (Figure 2C_i), myogenic (Figure 2C_ii) and glial (Figure 2C_iii) lineages, as shown by the positive immunolabeling against the lineage specific markers. These data are in accordance with previous findings (Carnevale et al., 2018; Pisciotta et al., 2018; Zordani et al., 2019).

### Stem cell morphology and proliferation of hDPSCs on PEDOT: PSS

**FIGURE 3:**
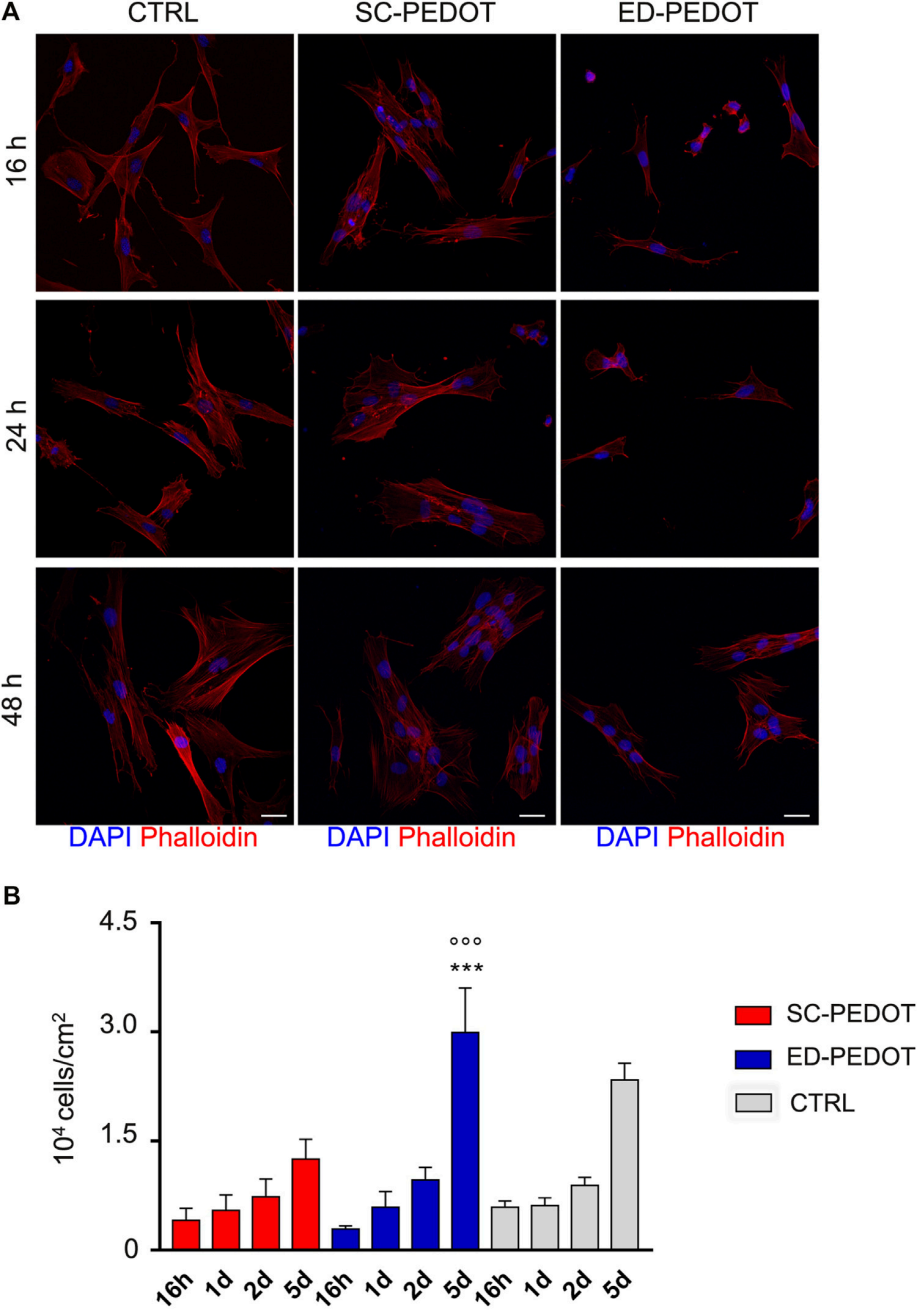
Evaluation of cell morphology, adhesion and proliferation of neural crest-derived stem cells on PEDOT:PSS films. **(A)** Immunofluorescence images showing cell morphology and adhesion of phalloidin-stained hDPSCs (red) grown on PEDOT:PSS films at different time points (16, 24 and 48 h). Nuclei were counterstained with DAPI. hDPSCs cultured on plastic culture plates were taken as controls. Scale bar: 20 μm. **(B)** Histograms represent cell proliferation of hDPSCs cultured on PEDOT:PSS films (16 h, 1, 2 and 5 days). Cell countings are reported as mean ± standard deviation (SD). ^***^ *p* < 0.001 hDPSCs cultured on ED-PEDOT *vs* hDPSCs cultured on SC-PEDOT, ***p < 0.001 hDPSCs cultured on ED-PEDOT *vs.* control hDPSCs; one-way ANOVA followed by Newman-Keuls post hoc test.

STRO-1^+^/c-Kit^+^ hDPSCs were seeded on PEDOT:PSS films, then cell adhesion and morphology were evaluated at 16, 24 and 48 h of culture (Figure 3). As revealed by Phalloidin stain (Figure 3A), hDPSCs adhered readily to both PEDOT thin films, as early as after 16 h. Notably, stem cells exhibited a fibroblast-like morphology when cultured on SC-PEDOT and plastic culture plates, whereas the culture on the ED-PEDOT surface showed a spindle-shaped morphology at earlier times of culture, i.e., 16 and 24 h. Regarding cell proliferation, no statistically significant difference was observed between the two thin films up to 2 days of culture. In contrast, a statistically significant increase in proliferation was observed on day 5 in hDPSCs grown on ED-PEDOT, when compared to the counterpart cultured on SC-PEDOT (***p < 0.001) and on plastic culture plates (***p < 0.001), respectively (Figure 3B).

### Evaluation of stemness and neural crest markers after culturing on PEDOT: PSS films

**FIGURE 4:**
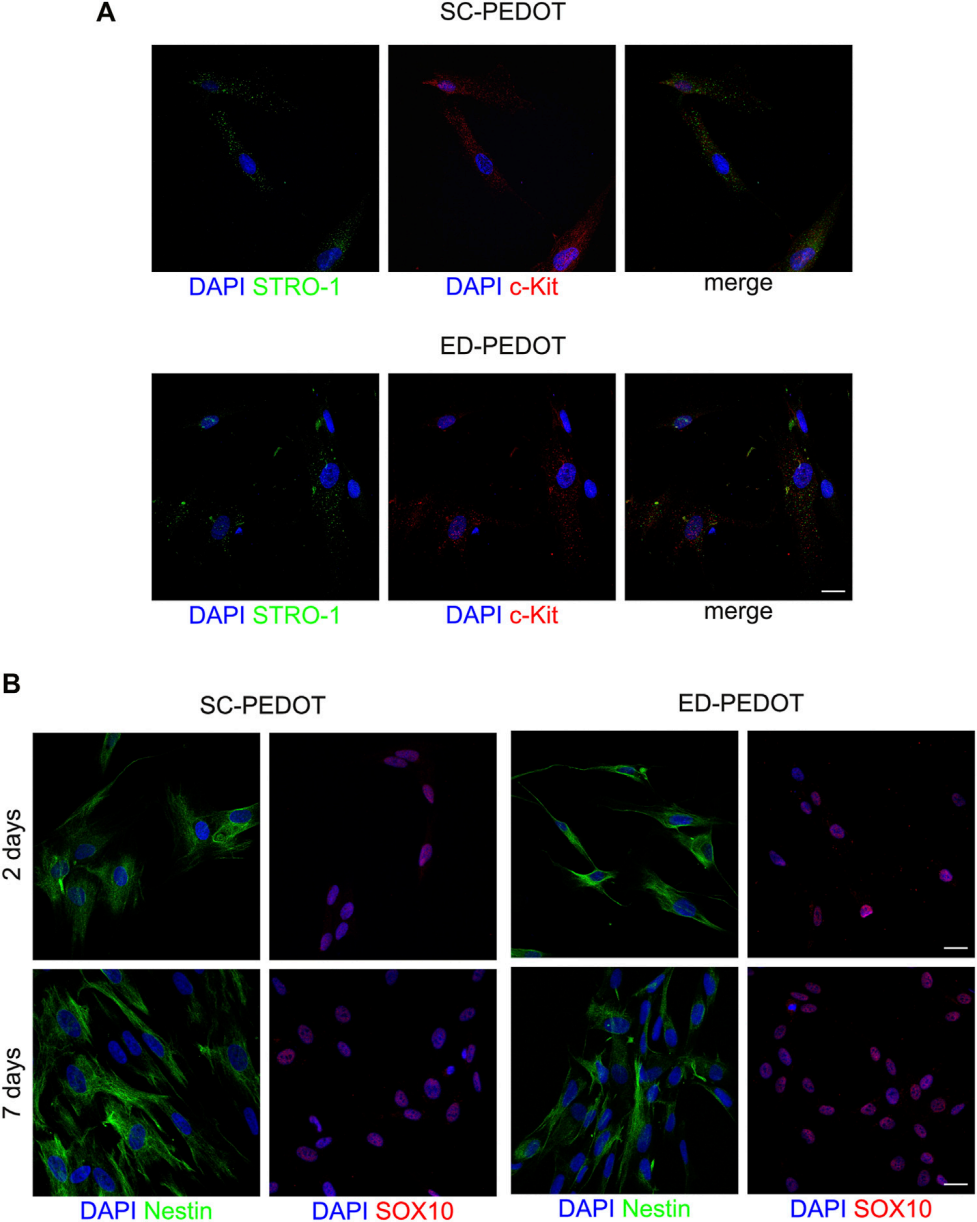
Evaluation of stemness and neural crest markers in hDPSCs cultured on PEDOT: PSS films. **(A)** Immunofluorescence images show the expression of the stemness markers STRO-1 (green) and c-Kit (red) after 48 h of culture and **(B)** the neural crest related markers Nestin (green) and SOX10 (red) in hDPSCs after 48 h and 7 days of culture on both types of PEDOT:PSS films. Nuclei were counterstained with DAPI. Scale bar: 10 μm **(A)**, 20 μm **(B)**.

After 48 h of culture, hDPSCs were analysed for the expression of the stemness markers STRO-1 and c-Kit (Figure 4). Immunofluorescence analysis revealed that hDPSCs labelled positive against both the markers, either on SC- and ED-PEDOT films (Figure 4A). Similarly, an intense immunostaining for the neural crest markers Nestin and SOX10 was observed, after 2 and 7 days of culture under both the experimental conditions (Figure 4B), thus suggesting that the PEDOT films did not affect the stemness phenotype of the immune-selected hDPSCs.

### Induction of neurogenic commitment in hDPSCs cultured on PEDOT: PSS

**FIGURE 5:**
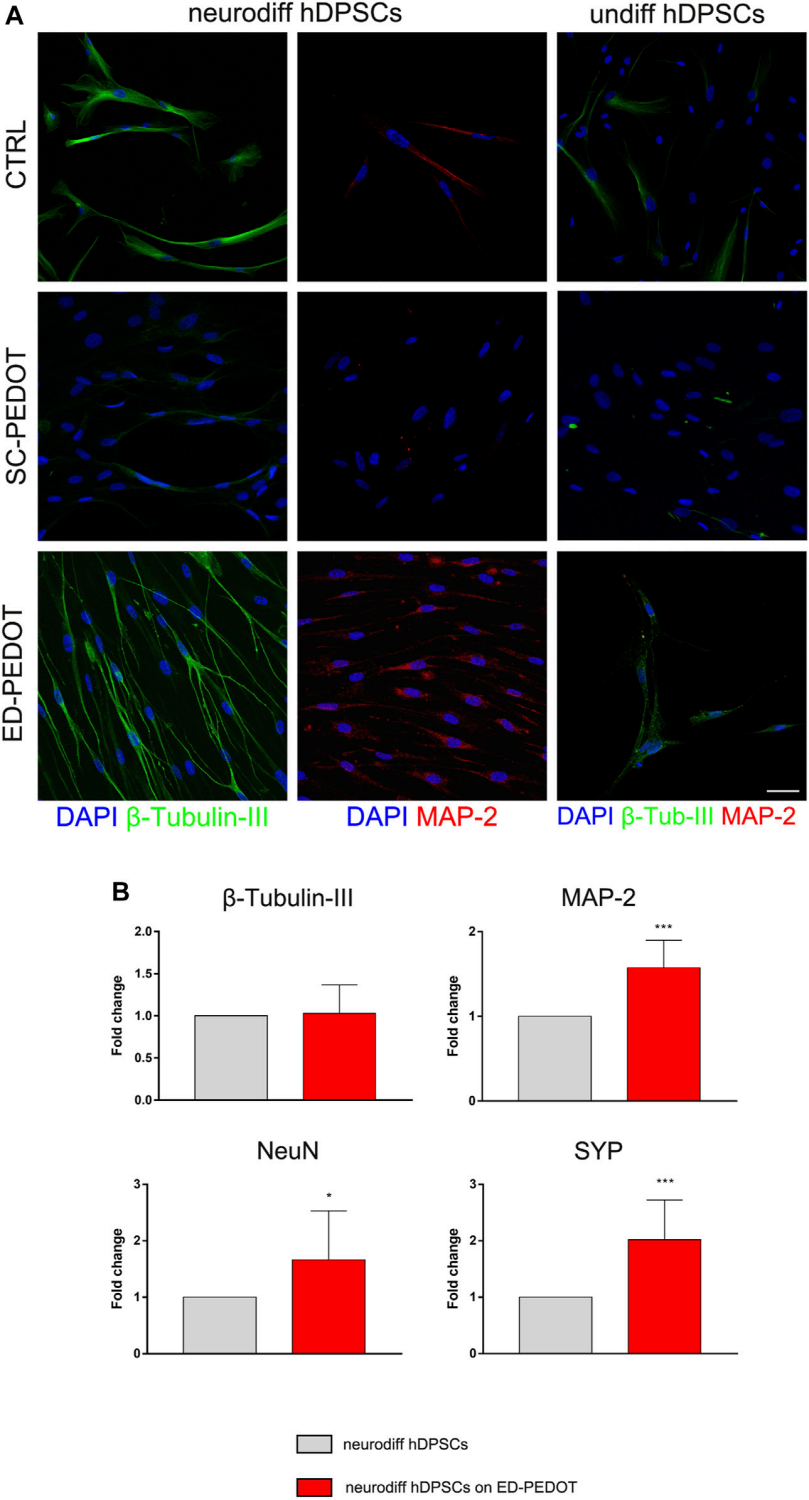
Evaluation of neurogenic differentiation of hDPSCs on PEDOT: PSS films. The expression of the neuronal markers β —Tubulin-III (green) and MAP-2 (red) in hDPSCs cultured in neurogenic medium for 7 days on both types of PEDOT:PSS films are shown by representative immunofluorescence images. Nuclei were counterstained with DAPI. hDPSCs differentiated on plastic culture plates were used as controls. Scale bar: 20 μm **(A)**. Real-time PCR analysis showing fold increase of mRNA levels of β —Tubulin-III, MAP-2, NeuN and Synaptophysin (SYP) in hDPSCs after 3 weeks of neurogenic induction on ED-PEDOT films. Data represent mean ± SD of fold change obtained from three independent experiments. ^*^ *p* < 0.05, ^***^ *p* < 0.001 vs. neurodiff hDPSCs (i.e., on plastic culture plates) **(B)**.

In order to evaluate the effects of PEDOT:PSS substrate (and in particular of the two different PEDOT:PSS layouts) on the capability of STRO-1^+^/c-Kit^+^ hDPSCs to commit towards the neurogenic lineage, cells were cultured under the appropriate inductive stimuli, for 1 week and 3 weeks, respectively (Figure 5). As reported in Figure 5A, hDPSCs differentiated on SC-PEDOT slightly labelled against β-Tubulin-III whereas MAP-2 immunostaining was not detected. In contrast, after 1 week of induction on ED-PEDOT, hDPSCs strongly expressed β-Tubulin-III, revealing a cytoskeletal re-organization and an appreciable morphology shift towards a neuronal-like shape (Figure 5A). Interestingly, a basal level of β-Tubulin-III expression was already detectable in undifferentiated hDPSCs grown on ED-PEDOT, similarly to control samples.

Neurogenic induction of hDPSCs on ED-PEDOT was also confirmed by positive labelling for MAP-2. Notably, as shown in Figure 5A, cells arranged themselves in a parallel alignment, when compared to their counterpart cultured on SC-PEDOT, as early as after 7 days of induction. Based on this evidence, hDPSCs were also differentiated for 3 weeks on ED-PEDOT, then Real Time PCR analyses were conducted. Data confirmed the achievement of the neurogenic commitment of hDPSCs, when cultured on ED-PEDOT for longer experimental times (Figure 5B). Histograms show a statistically significant increase in mRNA levels of MAP-2 and later neuronal markers NeuN and SYP, when compared to control hDPSCs (*p < 0.05, ***p < 0.001 vs. neurodiff hDPSCs; Figure 5B).

**FIGURE 6:**
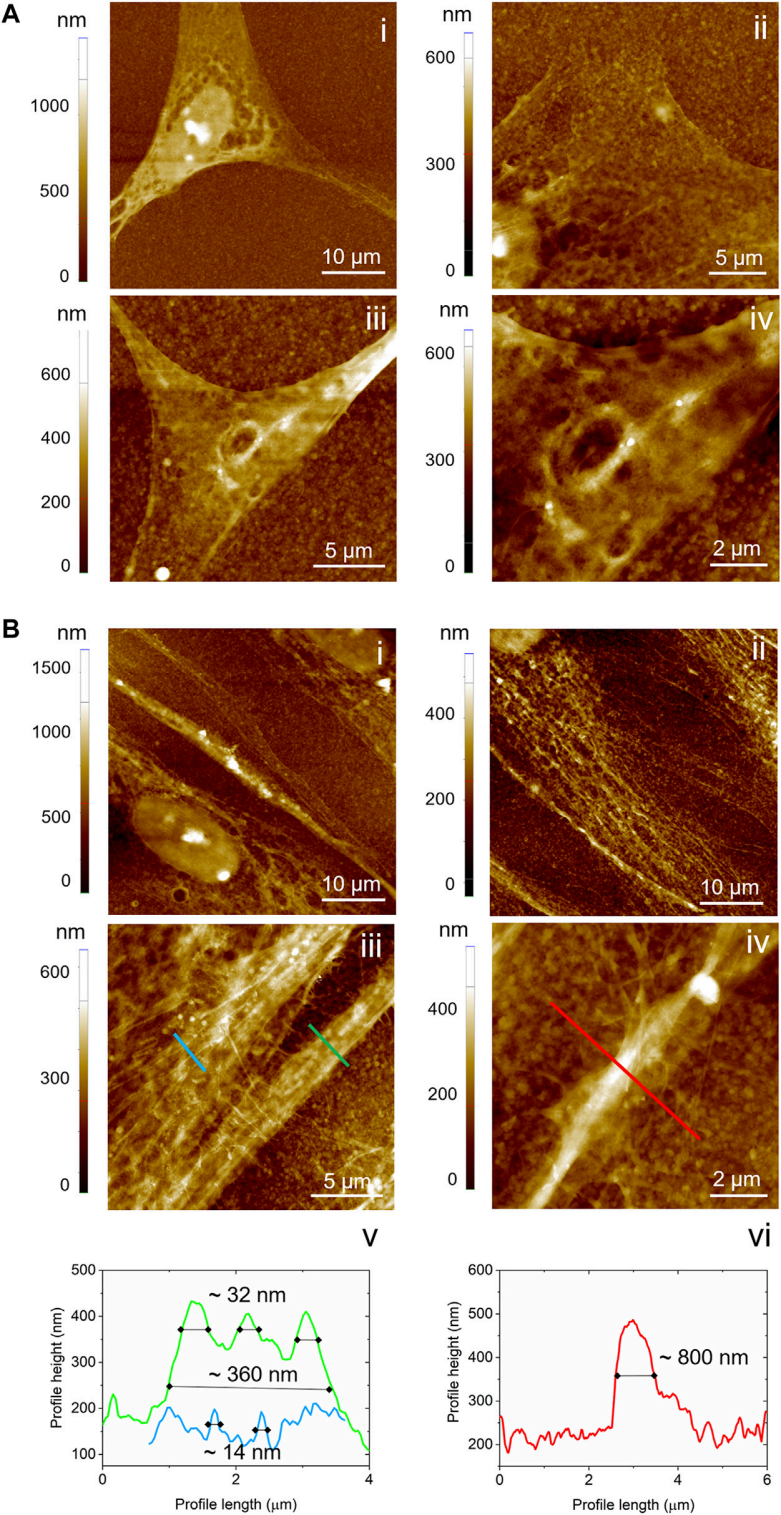
AFM analysis of cell morphology at the micro- and nanoscale. Topography images acquired on hDPSCs cultured on ED-PEDOT **(A)** before and **(B)** after 7 days of differentiation. In panels b, v and b, vi, the profile lines corresponding to the lines in b, iii and b, iv, are reported.

Further insights into the remodelling of the cytoskeleton of hDPSCs during early neurogenic differentiation time were obtained by imaging the adherent cells on ED-PEDOT with an atomic force microscope (Figure 6), as AFM allows the collection of morphological details that cannot be captured by conventional optical images. Topography AFM images show that undifferentiated stem cells exhibited a spindle-like morphology (Figures 6A_i,iii), appearing well adhered to the substrate, also due to intimate contact between the lamellipodia and the nanostructured PEDOT: PSS surface (Figures 6A_ii,iv), but without any obvious large scale arrangement of actin microfilaments and microtubules. In contrast, stem cells undergoing neurogenic induction were elongated along the same direction (Figures 6B_i,ii), showing abundant, well-developed and highly aligned microtubules, spreading from the perinuclear region and characterised by complex arrangements (Figures 6B_i–iv). Interestingly, the presence of different-diameter microtubules (from a few tens to several hundreds of nanometers, Figures 6B_iii–vi) as well as smaller-diameter filaments perpendicular to larger aligned microtubules (Figures 6B_iii) could be observed.

### Effects of PEDOT: PSS on the immunomodulatory phenotype of hDPSCs: FasL expression

**FIGURE 7:**
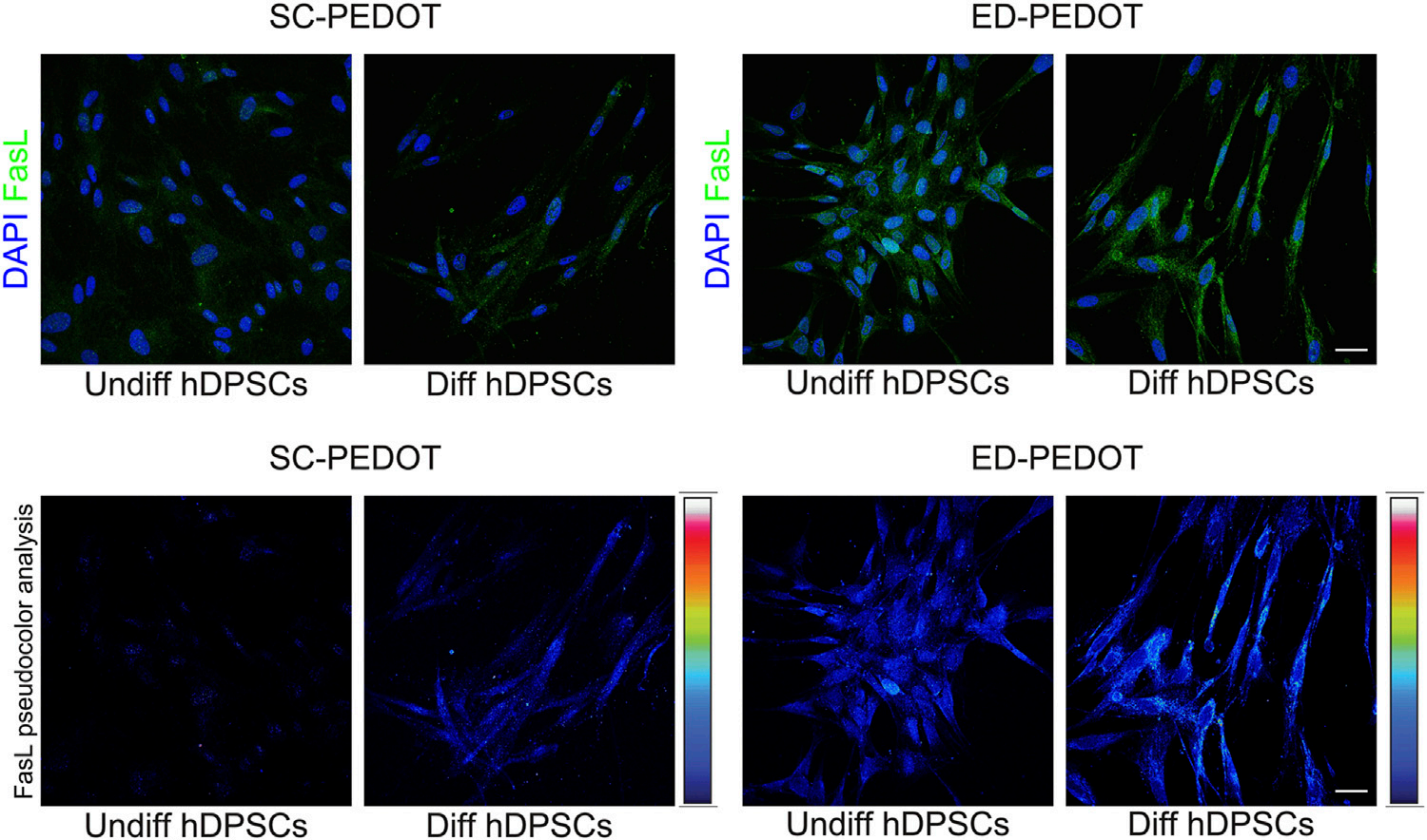
Evaluation of FasL expression in hDPSCs after culture on PEDOT: PSS films. Immunofluorescence (top) and pseudocolour (bottom) analysis of FasL immunolabeling is shown in hDPSCs after 7 days of culture in the neurogenic medium. Control group consisting in hDPSCs cultured for 7 days in standard expansion medium. Nuclei were counterstained with DAPI. Scale bar: 20 μm.

The expression of FasL was evaluated in hDPSCs cultured for 7 days in neurogenic induction medium, on both PEDOT:PSS films. As shown in Figure 7, culturing on SC-PEDOT revealed a weak immunostaining against FasL in neurogenic induced hDPSCs whereas almost no labelling was detected in undifferentiated hDPSCs. On the other hand, as highlighted by pseudocolour analysis, when stem cells were grown on ED-PEDOT, FasL expression was yet strongly detected under standard culture conditions with a more intense immunolabeling being observed after 1 week of induction towards neurogenic commitment, suggesting that ED-PEDOT supports the expression and maintenance of FasL under both expansion and differentiation conditions.

## Discussion

Addressing biocompatibility of PEDOT:PSS is of paramount importance for a variety of biomedical and biotechnological applications, including the development of stable and long-term effective microelectrodes for neural recording and stimulation or electroactive scaffolds for tissue engineering and repair. In the present study, we evaluated the behaviour of neural crest derived stem cells isolated from human dental pulp on PEDOT: PSS thin films, as this stem cell population represents a valuable source due to the peculiar stemness properties. In particular, we investigated cell behaviour on two prototypical PEDOT: PSS layouts, i.e., PEDOT thin films deposited from a commercial water dispersion or by electrochemical polymerisation. Whereas electrodeposited PEDOT: PSS is known to be generally not cytotoxic (Asplund et al., 2009; Asplund et al., 2010; Castagnola et al., 2015; Yazdimamaghani et al., 2015; Bhatt et al., 2016), that is not always the case for the water dispersions. Indeed, highly conductive formulations usually come with the presence of additives with questionable toxicity and often are available at very low pH values (Stříteský et al., 2018; Kim et al., 2018). In this study, we chose to investigate a low cytotoxic PEDOT: PSS grade, at the expense of its conductivity, as the addressing of the cellular behaviour upon electrical stimulation was beyond the scope of the present work.

Owing to the different deposition method, ED-PEDOT and SC-PEDOT thin films exhibited comparable thickness but different surface characteristics, being electrodeposited ones significantly rougher but only slightly more hydrophobic than the spin coated one. It is well known that both surface roughness and wettability are among the principal surface characteristics affecting protein adsorption (first) and cell adhesion (later on), and eventually proliferation and viability (Dowell-Mesfin et al., 2004; Tonazzini et al., 2010; Zareidoost et al., 2012; Chelli et al., 2014). In particular, biomaterial surfaces showing higher (random or defined) micro- and nano-roughness, are expected to promote cell growth compared to smoother surfaces (Klymov et al., 2013; Bianchi et al., 2016; Giusto et al., 2017; Graziani et al., 2019; Tian et al., 2022). Therefore, being the other material properties investigated here (i.e., wettability, sheet resistance and electrochemical impedance) comparable between the two groups, the higher proliferation of hDPSCs on ED-PEDOT compared to SC-PEDOT observed at day 5 can be ascribed, to a first approximation, to the rougher surface of the former. Clearly, other surface characteristics may influence cell adhesion and differentiation, including the net charge (or zeta potential) of the surface or the pH. Accordingly, the excess of negatively charged PSS^−^ may have negatively impacted the number of proliferating cells, as a negatively charged surface can better repel the negatively charged membrane of a cell compared to positively charged or zero net charge surfaces (Hallab et al., 1995; Metwally & Stachewicz, 2019). In addition, the excess of acid protons of PSS in SC-PEDOT films (pH of the initial aqueous dispersion was 2.4) could have lowered the pH in the proximity of the surface compared to ED-PEDOT, establishing a less favourable microenvironment for cell growth and proliferation.

In addition to the proliferation rate, the different surface characteristics between SC- and ED-PEDOT film also seem to influence the rearrangement of the cytoskeleton. Indeed, whereas hDPSCs seeded on SC-PEDOT showed a fibroblast-like morphology, cells on ED-PEDOT modified their cytoskeleton architecture, rearranging towards a more spindle-like morphology, which closely relates to their neural crest derivation. Nevertheless, when looking at the expression of the stemness markers, both SC-PEDOT and ED-PEDOT did not affect the expression of STRO-1 and c-Kit in hDPSCs. In addition, the neural crest markers Nestin and SOX10 were maintained, revealing that the different deposition method of PEDOT did not alter the stemness phenotype of neural crest derived hDPSCs.

We then focussed on the capability of PEDOT:PSS films to support neurogenic commitment of hDPSCs. Noteworthy, when neurogenic induction was performed by culturing hDPSCs under appropriate stimulus conditions, an early expression of β-Tubulin-III was observed, particularly prominent in cells cultured on ED-PEDOT. Beta tubulins are one of two structural components that form the microtubule network. While general tubulins play a role in a wide range of cellular processes, such as mitosis and motility, β-Tubulin-III is specifically localised to neurons and its expression correlates with the earliest phases of neuronal differentiation, with consequent implications in neurogenesis, axon guidance and maintenance (Mariani et al., 2015). In our study, as early as after 7 days of neurogenic induction, hDPSCs cultured on ED-PEDOT film not only showed a strong expression of this marker, but also displayed a morphological shift toward neuron-like cells with an aligned arrangement, in comparison to hDPSCs differentiated on SC-PEDOT and on plastic culture plates. Also, neurogenic differentiation of hDPSCs on ED-PEDOT induced the expression of MAP-2, a protein that is expressed primarily in neurons and plays a role in binding to and stabilising microtubules (Dehmelt and Halpain, 2005). Interestingly, the morphological transition of hDPSCs during differentiation from a spindle-like morphology towards an elongated one, may be also appreciated by AFM imaging. In particular, high resolution AFM images allowed us to appreciate the reorganisation of the cytoskeleton architecture showing an aligned remodelling of microtubules in differentiated cells. These findings support the evidence that not only the chemical composition of a scaffold but also its biophysical cues, such as micro/nanotopography and roughness, can affect stem cell behaviour including cell differentiation (Krishna et al., 2016). To this purpose, previous investigations on different micro/nanostructured biomaterials, such as carbon nanotubes (Shao et al., 2018), micropatterned polystyrene substrates (Recknor et al., 2006) and poly-ε-caprolactone scaffolds (Mohtaram et al., 2015), proved that the integration of biological, chemical and physical cues is crucial in guiding the neuronal differentiation of neural progenitor stem cells.

Noteworthy, data from the present study indicate that PEDOT:PSS films, in particular the electrodeposited ones, promoted this commitment at much earlier times (i.e., within 1 week of stimulation) besides supporting the achievement of a mature neuronal phenotype, as confirmed by the expression of late neuronal markers, such as NeuN and SYP, after 3 weeks of differentiation, in accordance with our previous studies (Pisciotta et al., 2018).

This suggests that PEDOT-based materials might be effective in maintaining the neural crest-related properties of hDPSCs in terms of stemness phenotype and neurogenic differentiation potential.

Finally, we remark that neural crest derived hDPSCs not only represent a valuable stem cell source for their typical stemness properties, classically defined by cell proliferation and multipotency, but also for their immunomodulatory properties. The latter have been under active investigation so far and hold a primary role in implementing the concept of stemness phenotype. Here, we evaluated the expression of FasL in hDPSCs undergoing neurogenic induction in order to evaluate for the first time the capability of PEDOT:PSS substrate to support the expression and maintenance of this immune-regulatory marker. Indeed, it is widely reported in literature that Fas/FasL pathway activation represents one of the multiple mechanisms through which stem cells are able to modulate the immune response (Zhao et al., 2012; Riccio et al., 2014; Pisciotta et al., 2020). It is also known that the constitutive expression of FasL in specialised tissues, i.e., eye, testis and nervous system, can be considered a key component for the maintenance of immune privilege of these tissues (Griffith et al., 1995; Green and Ferguson, 2001). It was demonstrated that neurons and glial cells are able to express FasL, with the consequent capability to limit and prevent inflammatory response and maintain immune tolerance conditions. In this study, we demonstrated that the expression of FasL was evident in hDPSCs differentiated on ED-PEDOT film, when compared to the counterpart stem cells grown and differentiated on SC-PEDOT. Again, these data are in compliance with previous evidence (Pisciotta et al., 2018) and confirmed the ability of neural crest derived hDPSCs to maintain the expression of FasL even when induced towards the neurogenic commitment. Notably, the present findings suggest that PEDOT-films provide suitable conditions for the maintenance of stemness and immunomodulatory properties of these neural crest derived stem cells.

## Conclusion

In this study, we investigated the behaviour of hDPSCs on both spincoated and electrodeposited PEDOT:PSS thin films, in order to catch possible analogies and differences between two of the most used PEDOT layouts in organic bioelectronics and tissue engineering. We demonstrated that electrodeposited PEDOT:PSS films are highly effective in preserving the stemness properties of neural crest derived hDPSCs, whose neurogenic potential and immunomodulatory phenotype appeared to be highly promoted by the PEDOT: PSS substrate. Data from the present study might provide the basis for further investigations aiming to study the effects of functionalized PEDOT: PSS films with neural crest derived stem cells secretion products, i.e., exosomes, for the study of novel regenerative approaches that may combine the immunomodulatory and anti-fibrotic effects of stem cells.

## Data Availability

The original contributions presented in the study are included in the article/supplementary material, further inquiries can be directed to the corresponding author.
